# Sustainable valorization of marine plastic residues via hydrothermal liquefaction for clean energy recovery

**DOI:** 10.1038/s41598-025-32471-3

**Published:** 2026-01-13

**Authors:** Mahadevan Vaishnavi, S. Raja, Maher Ali Rusho, Tesfaye Barza Zema

**Affiliations:** 1 Center for Advanced Multidisciplinary Research and Innovation, Chennai Institute of Technology, Chennai, 600069 Tamilnadu India; 2 Masters of Engineering in Engineering Management, Lockheed Matin Engineering Management, University of Colorado, Boulder, CO 80308 USA; 3 College of Engineering, Division of Mechanical Engimeering, Wolaita Sodo University, Soddo, 109-2244 Ethiopia

**Keywords:** Marine pollutant residue, Hydrothermal liquefaction. bio crude, Solid residue, Mixed waste management, Energy science and technology, Engineering, Environmental sciences

## Abstract

This study evaluates hydrothermal liquefaction (HTL) of real-world marine pollutant residues (MPR) composed of mixed plastics, organic matter, paper, and textiles. Using diatomaceous earth (DE) catalysis and aqueous-phase (AQ) recirculation, the effects on product yield, composition, and energy recovery were examined. Under optimized conditions (380 °C, 80 min, 10 wt% DE, RR = 6 mL g^-1^), a maximum bio crude yield of 51.6% with an HHV of 40.3 MJ kg^-1^ was achieved. Elemental, molecular, and thermal analyses (CHNS, GC–MS, FTIR, TGA) indicated improved hydrocarbon content and reduced oxygenation in the DE + AQ configuration. Net energy ratio (NER) calculations showed that the process can achieve energy-positive operation under conditions of elevated AQ recirculation temperature, highlighting the importance of heat integration. While these results demonstrate effective conversion of heterogeneous marine residues into energy-dense products, broader sustainability claims require further assessment of emissions, wastewater toxicity, and scale-up feasibility. The study provides experimentally grounded insights into HTL as a potential component of coastal waste valorization strategies.

**Supplementary Information:**

The online version contains supplementary material available at 10.1038/s41598-025-32471-3.

## Introduction

Global waste generation currently stands at 2.01 billion tonnes annually, with projections indicating a 70% increase by 2050 due to population growth and consumption patterns^1^. Among these wastes, polymeric materials pose significant challenges owing to their resistance to degradation and strong bonds with organic matter^2^. Plastic pollution, in particular, is one of the most critical environmental concerns of the current 21st century. In 2015, global plastic production reached a whooping 4.9 billion metric tons (MT), with an anticipated rise up to 12 billion MT annually by the year 2050^3^. Over 50% of these plastics contain hazardous monomers and additives, posing severe environmental risks^4^.

Approximately 25% of waste remains untreated, ultimately fragmenting into microplastics and infiltrating marine ecosystems. Annually, an estimated 14 MT of plastic enter oceans, significantly impacting marine biodiversity and ecological health^5^. Addressing this escalating challenge necessitates effective waste management strategies and innovations targeting polymer degradation to mitigate environmental and ecological risks. Once released into ecosystems, plastics undergo degradation, fragmenting into smaller particles and compounding waste management complexities^6^. Plastics are classified based on their size into four categories: macroplastics (> 25 mm), mesoplastics (varying from 5 to 25 mm), microplastics (from 0.1 μm to 5 mm) and nanoplastics (< 0.1 μm)^7^.

The micro plastics, particles less than 5 mm, are among the most pervasive pollutants globally, threatening ecosystem stability^8^. They are detected in diverse environments, including air, soil, oceans, freshwater systems, Arctic lake sediments, and wastewater. Their diminutive size and extensive surface area facilitate the adsorption of hazardous contaminants, pharmaceutical residues including polycyclic aromatic hydrocarbons, polybrominated diphenyl ethers and heavy metals, thereby contributing to chronic toxicity in biological organisms^9^. The widespread distribution of microplastics results from improper plastic disposal and inadequate waste management, with significant contributions from solid waste. Despite their substantial impact, microplastics in solid waste remain understudied compared to other sources. These pollutants persist in ecosystems, contaminating rivers, lakes, seas, sediments, and landfills, further exacerbating environmental degradation and posing risks to human and ecological health.

Plastic fragments, derived from industrial activities, the automotive sector, and municipal solid waste, combine with organic matter and are subsequently ingested by marine organisms. Plastics, particularly microplastics, in marine systems do not exist as isolated entities but rather in conjunction with organic marine matter, forming what is known as marine pollutant residue (MPR). The Adyar River estuary in Chennai, India, was selected as the source of marine pollutant residues (MPR) due to its well-documented accumulation of mixed anthropogenic debris arising from urban discharge, tidal intrusion, and estuarine mixing. The river receives a continuous load of plastics, textiles, biomass fragments, and municipal waste, creating a heterogeneous pollutant matrix that closely reflects real coastal contamination conditions. This environmental setting provides a representative and compositionally complex feedstock for evaluating HTL performance on realistic marine pollutants rather than idealized laboratory mixtures. The MPR encompasses the enduring remnants of contaminants, which typically include organic matter (such as decomposing plant and animal material), plastics, heavy metals, and chemicals. These pollutants accumulate within marine ecosystems, posing significant threats to aquatic life and disrupting the overall ecological equilibrium^10^. Thus, the processing of MPRs entails the segregation of their components and fractions to establish an efficient management approaches and scalable industrial process. A comprehensive framework for the management and treatment of MPR must account for the inherent variability of natural constituents and the heterogeneous composition and distribution of anthropogenic pollutants, including plastics and other synthetic contaminants.

Recent advancements in treatment and pollutant handling technologies highlight the potential of energy recycling from microplastic by converting these non-recyclable plastics into renewable energy in various forms like heat, electricity, biofuels like biohydrogen and bioethanol, hydrocarbons, gases, and char via several conversion routes such as physical, chemical, biological and thermal processes. These methods degrade plastic waste, converting it into energy-efficient products while addressing environmental concerns. Evaluations incorporating metrics such as carbon efficiency, cumulative energy demand, global warming potential and production costs reveal that these technologies can reduce marine plastic volume up to 89% annually and emission of greenhouse gas by 30%. However, despite their benefits, these processes require segregation of plastics from the marine pollutant residue and may release harmful gases such as CO_2_, SO_x_, and NO_x_, highlighting the need for advanced emission control technologies to mitigate secondary environmental impacts^11^.

In this regard, the thermochemical conversion processes like pyrolysis, combustion, liquefaction and gasification are key methods for producing eco-friendly bio crude by breaking down biomass and plastic waste. Among these, Hydrothermal Liquefaction (HTL) has gained attention for its cost-effectiveness and carbon recovery efficiency in converting complex feedstocks to sustainable renewables such as chemicals and biofuels^12^. HTL serves as a sustainable and environmentally favourable substitute for crude oil, producing ‘bio crude’ via the thermal decomposition and molecular restructuring of biomass macromolecules^13^. This process is particularly effective in converting challenging feedstocks, such as polyethylene (PE) plastic waste into valuable products^14^.

HTL of biomass serves as an energy intensification process, efficiently capturing 70 to 80% of the chemical energy in the form of oil products, which constitutes only 20 to 30 (wt%) of the original feedstock mass^15^. This makes HTL a viable approach for the chemical recycling or valorisation of plastics wastes from post-consumer state. Also, mixed textile wastes can undergo hydrothermal liquefaction (HTL) without requiring preliminary processes such as sorting, separation, or the removal of dyes, pigments, and coatings. This characteristic offers a significant advantage over other chemical recycling techniques^16,17^. Additionally, research has shown that combining artificial synthetic polymers with natural lignocellulosic biomass or biological molecules during co-liquefaction can lower the reaction temperature of the polymers and create a synergistic effect that enhances bio crude production^18,19^. HTL is considered more advantageous than other thermochemical processes because of to its capability of operating at lower temperatures and pressures, offering higher yields and better energy efficiency, making it a promising technology for WTE applications^20,21^.

This study evaluates hydrothermal liquefaction (HTL) of real, field collected marine pollutant residues (MPR), representing a heterogeneous mixture of plastics, textiles, biomass, paper and fibrous contaminants. Unlike prior HTL studies that predominantly process only the plastic fraction of marine debris^22–24^, or in limited cases plastic and algae mixtures^25,26^, the present work examines the full pollutant matrix without separation, thereby capturing the true chemical and physical complexity of coastal waste streams. The study further introduces the combined application of diatomaceous earth (DE) catalysis and aqueous phase (AQ) recirculation, a synergy not previously investigated for realistic marine residues. Additionally, a multi scenario Net Energy Ratio (NER) analysis is conducted to quantify how varying recirculated phase temperatures influence the external heat duty, an aspect absent from existing marine debris HTL literature. Together, these contributions provide new insights into catalytic behaviour, solvent mediated reactions and system level energetics for complex, environmentally weathered marine waste, supporting the development of practical valorization pathways for coastal pollution. The findings offer a potential scalable perspective for converting non-recyclable pollutants into energy dense bio crude, contributing to Sustainable Development Goal 7 (Affordable and Clean Energy) and Goal 14 (Life Below Water), and reinforcing the potential of circular economy practices for coastal waste management.

## Materials and methods

This work involves obtaining marine pollutant wastes and converting them into valuable products through HTL process and characterising the yielded products. The detailed methodology involved in this work is briefed as follows:

### Waste sample collection

Marine pollutant residues (MPR) were collected from the Adyar Riverbank in Chennai, India (80°16′14.52″ E), near its confluence with the Bay of Bengal. This estuarine zone is known to accumulate mixed anthropogenic debris due to tidal intrusion and urban discharge, providing a representative sampling environment for heterogeneous coastal pollutants. Sampling was carried out on in June, strategically timed to precede the onset of the seasonal monsoon, during low tide conditions to capture surface accumulated residues. Sampling was carried out in June (pre-monsoon) during low tide conditions to preferentially collect surface accumulated residues. Ambient temperature at collection was 30 °C. In total, 1.5 kg of material was collected, homogenized, and subsampled. Samples were transported to the laboratory within 4 h of collection and stored at 4 °C for no longer than 48 h prior to processing. All water and debris samples were collected from publicly accessible riverbank locations and not part of any protected or restricted area. According to local municipal guidelines, no special governmental or institutional permit was required for sampling.

#### Water samples (WS)

A total of nine samples, each weighing 100 g, were collected from three designated sites along the Adyar River. The collection process involved the use of a 10-liter stainless steel bucket and a 0.3 mm mesh sieve (Haver Standard Test Sieves). Post-washing and separation, approximately 30 g of waste per litre was recovered, aligning with earlier reported findings^27^. The processed residues which were securely sealed in glass beakers covered with aluminium foil and further analysis was carried out in the lab. The samples were designated as WS1 through WS9 and were consistently referenced as such throughout all subsequent experiments in this study.

#### Coastal sediment samples (SS)

To obtain representative waste samples, six sediment samples were collected from the surface layer, excluding metallic components, using a sanitized stainless-steel spoon. Two samples per site were collected within a 1-meter radius to account for spatial variability, yielding a total of 100 g per site.

The collected waste samples were then securely transported to the laboratory for detailed analysis. These samples were designated as SS1 through SS6 and were consistently referenced as such throughout all subsequent experiments in this study.

#### Characterisation of water and sediment samples

Prior to separation of samples, it is important to note that, all the collected residues are influenced by brackish and marine conditions, as supported by earlier studies reporting salinity levels ranging from 5 to 25 ppt in the Adyar estuary zone^28,29^. While direct salinity measurements were not conducted in this study, the presence of marine-derived salts such as Na⁺, Cl⁻, Mg²⁺, Ca²⁺, and SO₄²⁻ can be reasonably inferred from works of^29,30^.

Before initiating the experiments, all acquired WS and SS were subjected to a preliminary process of screening to eliminate materials like glass, hazardous (e.g., batteries and electronic waste) and inert (e.g., metals) substances. The remaining fraction, deemed usable, was subjected to further analysis. The screened solid waste predominantly consisted of textile materials, plastics, paper, cardboard, decaying roots, plant matter, rubber, leaves, straws, and plastic containers of varying shapes, sizes, and weights. To facilitate analysis, the samples were categorized into five primary fractions: plastic covers, paper, textiles, plastic fragments, and organic waste. Each waste subcomponent was weighed individually and quantified using Eq. (1) in terms of wt% of the total weight for further experimental investigations.

1 $$\:Target\:component\:(wt.\:\%)=\frac{Weight\:of\:target\:component\:in\:sample}{Total\:weight\:of\:waste\:sample}*100$$

#### Representativity and elemental considerations

Due to the inherently heterogeneous nature of marine plastic residues (MPR), efforts were made to ensure representativity across all analytical procedures. Prior to characterization, the bulk collected residues were thoroughly homogenized through manual mixing, and triplicate sub-samples (~ 5–10 g) were drawn for elemental and proximate analyses. This ensured that the measurements reflected average compositional behaviour rather than isolated fragments. The relatively small quantity required for CHNS, FTIR, and FESEM-EDS analyses is a standard constraint in materials characterization, but was addressed here through sample replication and careful preparation. While direct quantification of halogens (Cl), especially Cl in plastic-rich marine residues, is recognized and its effects on product composition and corrosion are expected to be within operational tolerance, given the use of stainless steel reactors (316 L grade) and the absence of catalytic fouling or abnormal yields^31–33^. Future studies will incorporate XRF or ICP-OES for more detailed elemental speciation to better understand the long-term implications of these elements on catalyst life and reactor integrity.

### Experimental procedure

**Fig. 1:**
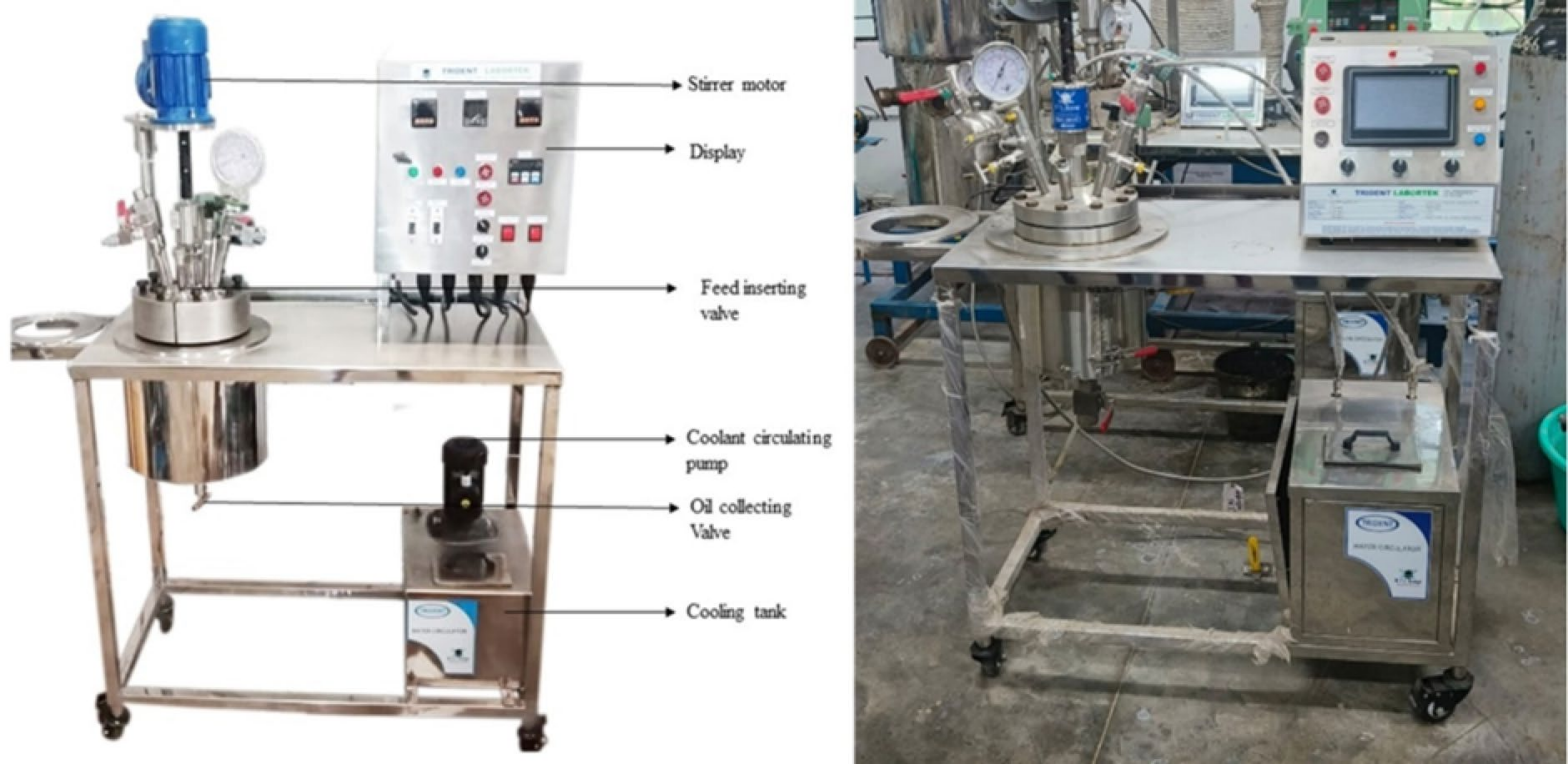
Experimental setup of HTL reactor: (**a**) layout and (**b**) photographic view.

The experiments of Hydrothermal Liquefaction (HTL) process were conducted in a high-pressure stainless-steel autoclave reactor with a 5 L capacity (Trident Labortek Pvt. Ltd.) represented in Fig. 1. The reactor was equipped with an integrated heater capable of maintaining temperatures up to 450 °C and a pressure tolerance of up to 25 MPa. The system included a magnetic stirrer with a maximum rotational speed of 1200 rpm to ensure uniform heat distribution. Additional features comprised a precision pressure gauge, a thermocouple for accurate temperature monitoring, a pressure relief valve for safety, and a coolant circulation pump for effective thermal regulation within the reactor chamber.

For each experimental run, 100 g of MPR was introduced into the reactor chamber, along with 1000 ml of distilled water. To ensure an inert reaction environment, nitrogen gas was used to purge the reactor which effectively eliminates oxygen to prevent oxidative reactions. The contents of the HTL reactor were heated at a controlled rate of 10 °C/min till the desired final operating temperature was arrived. This temperature was maintained for the specified residence time, after which the reactor was allowed to cool to ambient conditions to facilitate product collection. The experiments investigating the influence of operating temperature and residence time were conducted within the ranges of 300 °C to 400 °C and 40 to 120 min, respectively. As the feedstock utilized in this study consists of a complex mixture predominantly comprising plastics, which demand more harsh conditions for complete thermal degradation, the investigation focuses on higher temperature ranges and extended residence times.

Further, to investigate the HTL product distribution catalytic – HTL conditions, diatomaceous earth powder at varying weight ratios of 2.5–12.5 wt% were introduced into the reactor chamber, prior to commencement of the process. Also, during the testing of recirculation conditions, the aqueous phase (AQ) was introduced along with distilled water in varying ratios of 2–10 ml/g, and the reaction was proceeded as before. Upon completion of each experimental run, the HTL reactor was deactivated and allowed to cool naturally to ambient temperature. Subsequently, the pressure was safely released through the pressure relief valves, and the resultant mixture was collected for analysis. This procedure was repeated under identical conditions for the remaining 13 samples, yielding 28 mixtures in total, which were systematically analysed in this study.

### HTL product analysis

The products that are yielded from the HTL process of MPR feedstock are characterised in terms of various analysis as detailed below.

#### Quantitative analysis

From the liquid product, a 10 ml aliquot of the mixture was taken and filtered using Whatman No. 1 chromatography paper to eliminate particulates. The filtered sample was then transferred to a separation chamber and combined with an equal volume of dichloromethane (DCM). The mixture was permitted to settle down for a period of 24 h to facilitate phase separation into the aqueous phase (AQ) and bio crude. After separation, the two liquid layers were exposed to ambient conditions for 24 h to allow the evaporation of residual DCM. Based on density differentials, the AQ phase was identified as the bottom layer, while the bio crude phase formed the upper layer. The stratification of the two phases enabled their precise identification and subsequent analysis. Equation (2) was used to determine the target product yield, which is expressed as the ratio of the target product phase’s total mass to the MPR feedstock’s total mass.

2 $$\:Product\:yield\:\left(\:\%\right)=\frac{mass\:of\:product\:\left(g\right)}{mass\:of\:MPR\:feedstock\:\left(g\right)}*100$$

#### Qualitative and compositional analysis

The carbon, hydrogen, and nitrogen content in the feedstock and liquefaction oil was determined by employing an ultimate analyser and an elemental analyser, in accordance with standardized methods ASTM E871-82 (2006) and ASTM E1755-01 (2007). All CHNS elemental compositions reported in this study are expressed on a dry basis. The oxygen content was obtained from the difference between the total and the measured elemental components, providing a comprehensive elemental profile of the crude. The higher heating value (HHV) of the entire oil (in MJ/kg) is calculated using Dulong formula as given below^34,35^:

3 $$\:\mathrm{H}\mathrm{H}\mathrm{V}=\:0.335\mathrm{C}\:+\:1.423\mathrm{H}\:-0.154\mathrm{O}\:-0.145$$

where C, H, and O represent the mass of carbon, hydrogen and oxygen respectively. Also, the percentages of carbon recovery from the bio crude and solid residue was estimated as follows^36,37^:

4 $$\:Carbon\:recovery\:\left(\%\right)=\frac{\:C\:in\:product\:(wt.\:\%)\:}{C\:in\:MPR\:feedstock\:(wt.\:\%)}*Product\:yield\:\left(\%\right)$$

The energy recovery percentage of bio crude and solid residue were arrived by the Eq. (5)^38,39^.

5 $$\:Energy\:recovery\:\left(\%\right)=\frac{HHV\:of\:product\:\left(\frac{MJ}{kg}\right)\:}{HHV\:of\:MPR\:feedstock\:\left(\frac{MJ}{kg}\right)}*Product\:yield\:\left(\%\right)$$

Further, the composition of the bio crude and aqueous phase was analysed using Gas Chromatography-Mass Spectrometry (GC-MS), with Fourier Transform Infrared Spectroscopy (FTIR) employed to confirm the functional groups present in the bio crude. Liquid product composition was analysed utilizing an auto-injector (Agilent 7683 A) fitted Agilent 7890 GC system and a Flame Ionization Detector (FID). For this, 1 µL of the sample was injected into the system, with 99.9995% pure helium being employed as a carrier gas. The column temperature was initially set at 50 °C, subsequently ramped to 300 °C at the rate of 10 °C/min and held at the final temperature for 5 min.

Furthermore, Fourier Transform Infrared (FTIR) analysis, conducted using a PerkinElmer FTIRC 100,566 instrument (UK), was employed to identify and characterize the primary constituents in solid residue samples. This was achieved by examining the absorption peaks corresponding to functional groups within the spectral scale of 400–4000 cm⁻¹. Also, in addition to above, Shimadzu Thermogravimetric Analyzer (50 H) was used to conduct the Thermogravimetric analysis (TGA) on the MPR feedstock and the obtained bio crude by maintaining a nitrogen atmosphere at a heating rate of 10 °C/min. Approximately 20 mg of the sample was placed into the furnace, and the temperature was steadily increased from 100 °C to 800 °C, with the corresponding weight loss being recorded. The ASTM 7169 standard was adopted to assess the boiling point ranges of the various fractions of the bio crude^38,40^. The TGA sample corresponded to the same homogenized composite feedstock used for all HTL experiments, ensuring that the thermal degradation behaviour reflects the actual HTL feed material. Thermogravimetric analysis was performed in triplicate on this homogenized sample to assess reproducibility. All three curves exhibited consistent degradation patterns; therefore, a representative curve is reported in the Results section.

## Results and discussion

### Characterisation of feedstock (WS and SS) samples

**Fig. 2:**
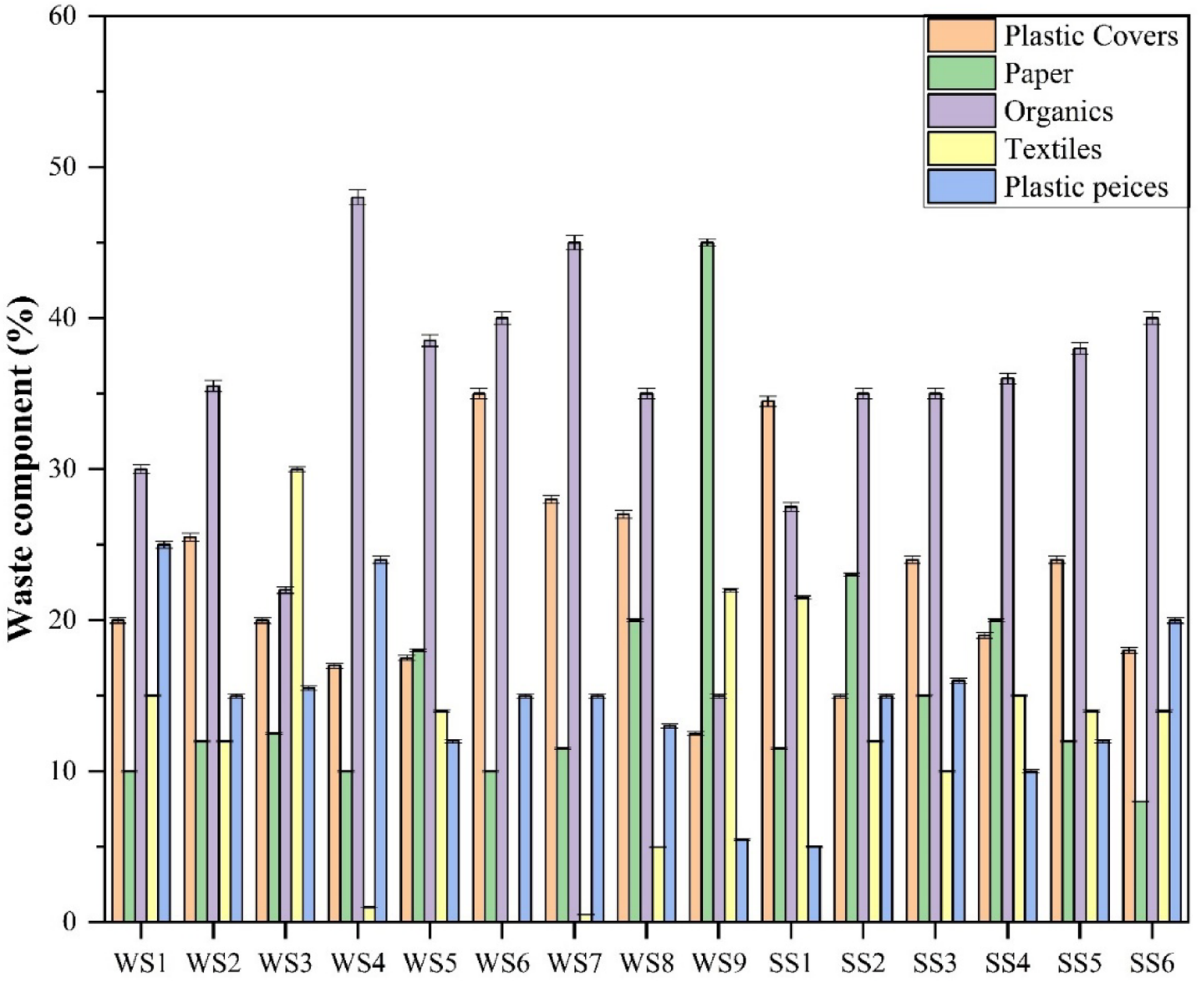
Proportion of wastes in various Water Sample (WS) and Sediment Sample (SS) samples.

**Fig. 3:**
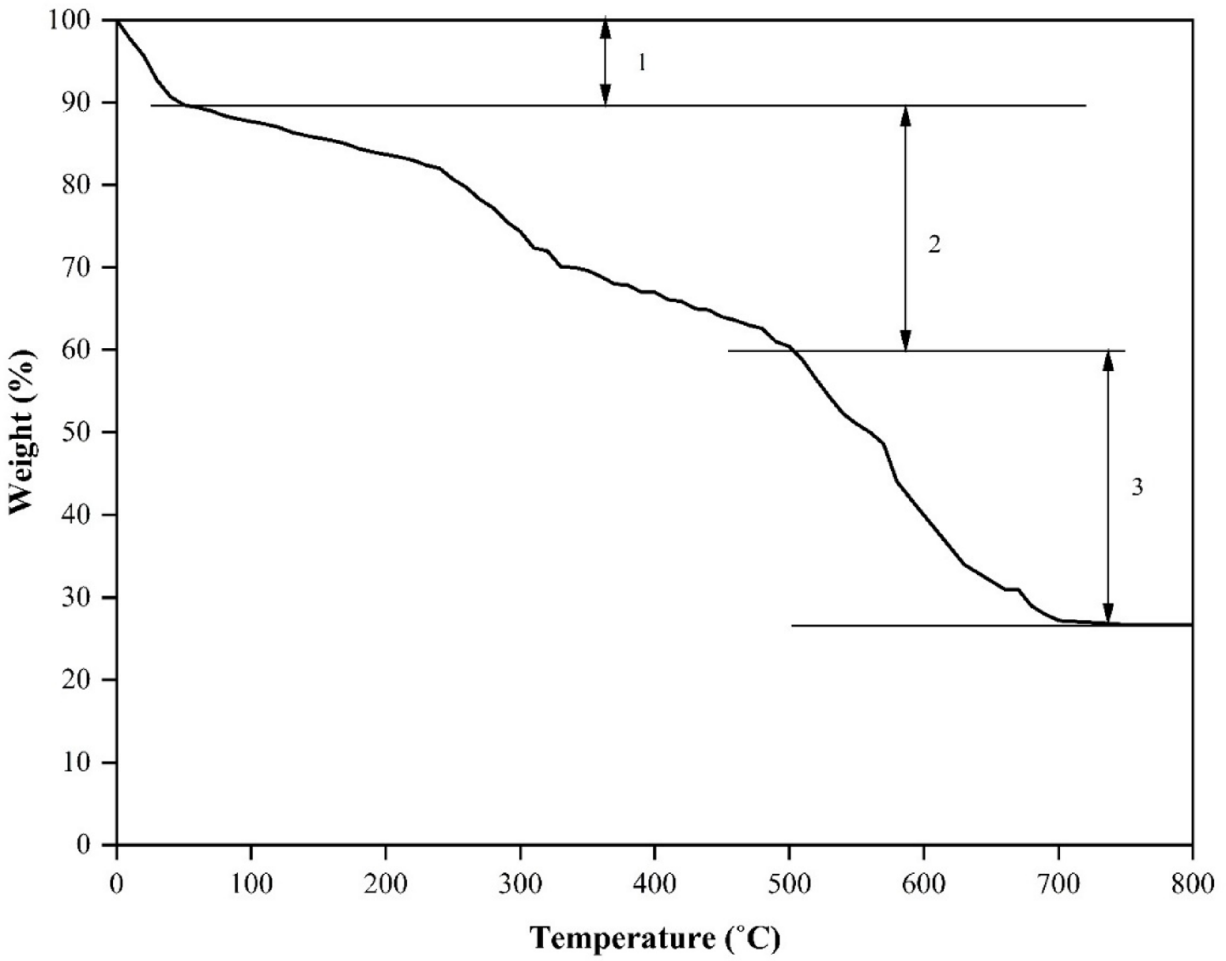
TGA curve of Marine Pollutant residue (MPR) sample.

The samples, labelled WS1–WS9 and SS1–SS6, were subjected to a screening process, and the outcomes are shown in Figs. 2 and 3. Among the nine samples (WS1 to WS9) collected from three sites, plastics emerged as the dominant fraction, with proportions varying across samples: 50% in WS6, 45% in WS1, and 43% in WS7. Within the plastic fraction, plastic bags/covers were most prevalent in WS6 and WS7, accounting for 70% and 65.11% of the total plastic content, respectively. In contrast, in WS1, plastic pieces contributed slightly more than plastic bags. Also, organic waste constituted the second most abundant fraction overall, with WS4 containing the highest organic content at 48%, followed by WS7 and WS6 at 45% and 40% respectively. Additionally, textiles and paper wastes individually contributed up to 20% of the total composition across most samples, except for WS9 and WS3, which had significantly higher proportions of paper (45%) and textiles (30%), respectively. Thus, the collected waste samples exhibited the highest plastic content, aligning with findings reported in a previous study by^41^.

In comparison to floating waste, the sediment contained a higher concentration of residues. The waste materials had an average size of 3 to 5 mm, with a maximum height of 10 cm, as illustrated in Fig. 2.

The weight of the waste was also analysed and recorded. The quantity of waste in the sediment varies with water flow, increasing during waste accumulation in the monsoon months. However, an average of 25 g/m² of sediment waste was observed under relatively low moisture conditions, as the samples were collected prior to the onset of the monsoon. Thus, in the SS samples, plastics constituted the dominant fraction. The highest plastic content was observed in SS3 (40%), followed by SS1 (39.5%) and SS6 (38%). Within the plastic category, plastic covers/bags consistently represented a higher proportion than plastic pieces across all SS samples. The organic fraction in the SS samples ranged from 35% to 40%, except for SS1, which contained 27.5%. As with the WS samples, the textile and paper fractions contributed slightly less than 25% in all SS samples.

Further, Table 1 summarizes the CHNS elemental analysis of the feedstock (MPR) across all water (WS1 to WS9) and sediment (SS1 to SS6) samples, highlighting the influence of the contents of C, H and O on feedstock type and quality. The MPR feedstock demonstrates a significantly higher content of carbon and a comparatively a lower oxygen content, which are on par with or slightly lesser than those found in the majority of HTL feedstocks namely microalgae like *Scenedesmus obliquus*^42^, Scenedesmus sp^43^. ,*Scenedesmus abundans*^44^ and household waste^45^. The increased carbon content observed in both WS and SS samples is predominantly because of the presence of plastic waste and organic constituents. Among the samples, WS6 and SS1 demonstrated the highest carbon content at 72.80% and 70.90%, respectively, along with the highest hydrogen content of 15.78% and 10.20%. Conversely, these samples exhibited the lowest oxygen content, with values of 10.13% for WS6 and 18.67% for SS1.

Overall, WS samples displayed increased C and H levels and lowered O content vis-à-vis SS samples. The lowest C and H levels were observed in WS9 (55.36% and 9.19%) and SS2 (59.03% and 8.56%), respectively, which also showed the highest oxygen content at 34.00% and 32.24%.

**Table 1:** Elemental analysis (CHNS) values, H/C, O/C and HHV for WS and SS samples.

Sample	C	H	*N*	S	O	H/C	O/C	HHV (MJ/kg)
WS1	65.05 ± 1.06	10.73 ± 1.12	1.05 ± 0.16	0.49 ± 0.02	22.68 ± 1.88	1.98	0.26	33.42 ± 2.12
WS2	67.79 ± 2.10	11.18 ± 1.05	1.09 ± 0.13	0.50 ± 0.01	19.44 ± 1.80	1.98	0.22	35.48 ± 2.01
WS3	63.12 ± 0.98	10.35 ± 0.97	1.86 ± 0.11	0.41 ± 0.03	24.26 ± 1.65	1.97	0.29	31.99 ± 1.99
WS4	72.35 ± 1.12	12.41 ± 0.99	1.12 ± 0.25	0.50 ± 0.01	13.62 ± 1.02	2.06	0.14	39.65 ± 2.06
WS5	64.50 ± 1.25	10.55 ± 1.03	1.24 ± 0.22	0.65 ± 0.01	23.06 ± 2.03	1.96	0.27	32.92 ± 1.92
WS6	72.80 ± 2.09	15.78 ± 1.19	1.19 ± 0.19	0.10 ± 0.02	10.13 ± 1.85	2.60	0.10	45.14 ± 2.15
WS7	68.02 ± 1.99	11.99 ± 1.22	1.10 ± 0.20	0.20 ± 0.02	18.69 ± 1.88	2.12	0.21	36.83 ± 1.82
WS8	65.50 ± 1.87	10.55 ± 1.02	1.05 ± 0.21	0.40 ± 0.03	22.50 ± 0.99	1.93	0.26	33.35 ± 2.78
WS9	55.36 ± 1.45	9.19 ± 0.89	1.10 ± 0.14	0.35 ± 0.01	34.00 ± 1.18	1.99	0.46	26.24 ± 2.25
SS1	70.90 ± 1.44	10.20 ± 1.11	0.13 ± 0.06	0.10 ± 0.02	18.67 ± 1.11	1.73	0.20	35.25 ± 2.16
SS2	59.03 ± 0.89	8.56 ± 0.87	0.15 ± 0.09	0.02 ± 0.01	32.24 ± 1.36	1.74	0.41	26.85 ± 2.09
SS3	68.78 ± 1.50	9.90 ± 0.82	0.22 ± 0.10	0.01 ± 0.00	21.09 ± 1.22	1.73	0.23	33.74 ± 1.93
SS4	60.12 ± 1.67	8.84 ± 0.96	0.58 ± 0.08	0.02 ± 0.01	30.44 ± 1.99	1.76	0.38	27.89 ± 2.15
SS5	67.05 ± 0.97	9.12 ± 1.16	1.01 ± 0.11	0.05 ± 0.01	22.77 ± 1.82	1.63	0.25	31.79 ± 2.22
SS6	65.50 ± 1.23	9.02 ± 1.00	1.13 ± 0.13	0.12 ± 0.02	24.23 ± 1.65	1.65	0.28	30.90 ± 2.89

Across all samples, nitrogen (N) and sulphur (S) content remained minimal, ranging between 0.1% and 1.86%. Based on C, H, and O content, the H/C ratios ranged from 1.63 to 2.60, with the highest values recorded in WS6 (2.60) and SS4 (1.76). Similarly, the O/C ratios were lowest for WS6 (0.10) and SS1 (0.20). The HHV spanned from 26.24 to 45.14 MJ/kg, with WS9 and WS6 exhibiting the lowest and highest values, respectively.

The compositions presented in this study offer a broad overview of the MPR constituents; however, it is important to note that the representative sample was formulated by averaging the proportions of the constituents. Although this sample is reflective of the MPR characteristics in the specified region, variations ranging from mild to severe may occur n MPR composition and must be accounted to optimize the performance of the proposed energy conversion technology. Furthermore, the composition outlined is specific to this region and should not be generalized to represent the overall intensification of marine pollutants globally. Therefore, for subsequent investigations, the MPR feedstock was systematically formulated as 40% plastics, comprising 23% plastic covers and 17% plastic fragments, 15% paper, 35% organic matter, and 10% textiles. This formulation was derived by averaging the constituent proportions from both water and sediment samples, ensuring a representative and balanced composition for further analysis.

Furthermore, TGA was used to evaluate the thermal stability of the feedstocks as the functions of temperature, which is represented in Fig. 3. This study centres on the thermal degradation behaviour of the feedstocks under a constant heating rate, aiding in the identification of distinct phases (1 ,2 and 3) within the HTL process for further investigation. The TGA curve distinctly illustrates the thermal breakdown of the MPR components across the evaluated temperature range. The initial loss of mass up to 80 °C, designated as region 1, corresponds to the water trapped due to evaporation within the sample. A significant reduction in weight in region 2, between 200 and 520 °C (peak at 325 °C), is ascribed to the decomposition of organic components, textiles, and paper fractions. Subsequently, in region 3, the weight loss noted in the temperature range of 550 to 650 °C (peak at 450 °C), is associated with the thermal breakdown of plastic waste constituents found in the MPR, driven by thermal energy^46,47^. Additionally, the heterogeneousness of the MPR sample subscribes to the relatively high solid residue yield post-TGA analysis (26.71 wt%). Thus, the TGA analysis revealed that the biomass degradation is initiated within the temperature limits of 250–320 °C, with approximately 40% of the material undergoing decomposition between 360 °C and 380 °C.

### Influence of operating parameters on HTL performance

The HTL process is influenced by several critical parameters that affect both the yield and quality of bio crude, including temperature, reaction duration, catalyst type, and the ratio of feedstock-to-solvent. The effects of these various operating parameters on HTL products are discussed here.

**Fig. 4:**
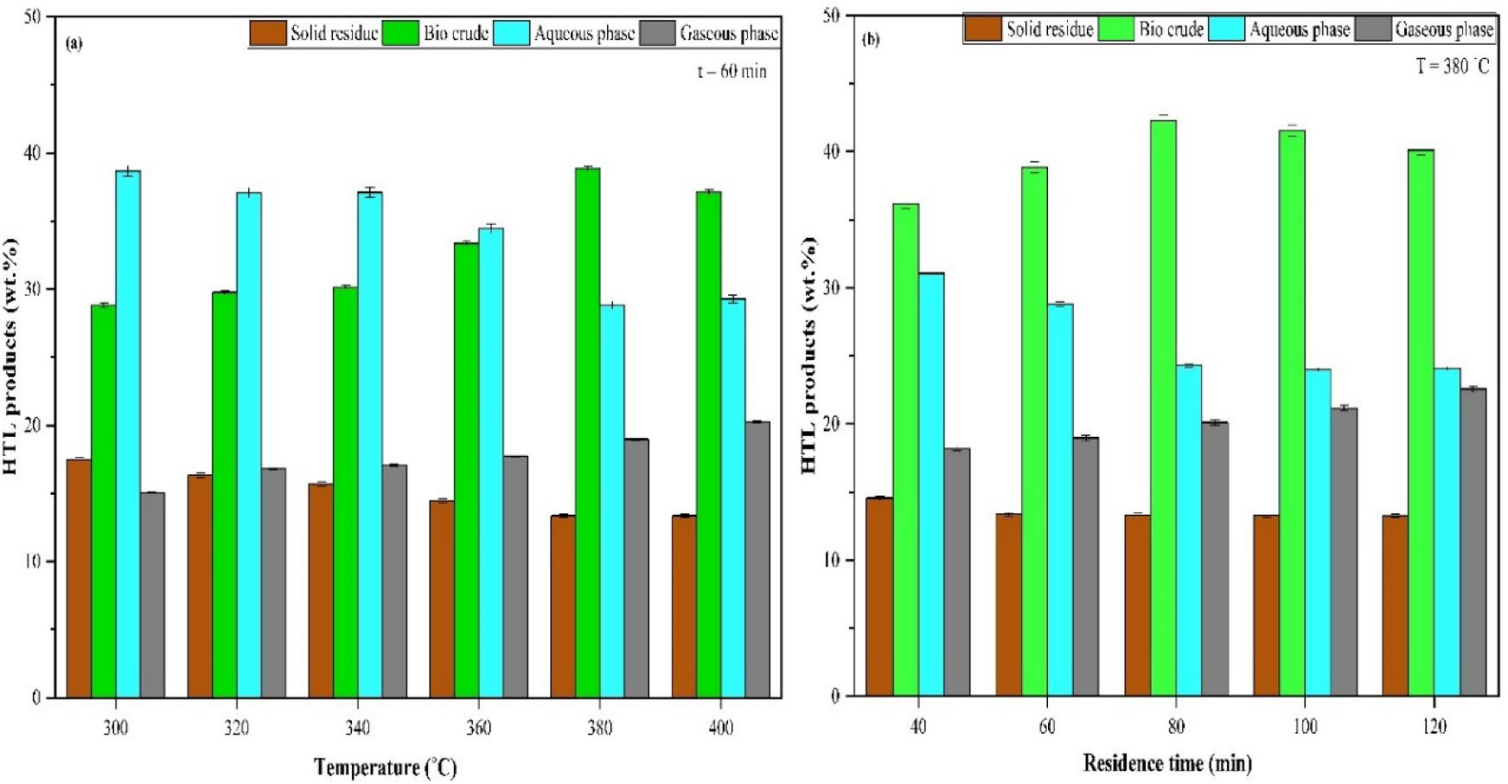
(**a**) Effect of temperature (300 °C to 400 °C) on HTL product distribution. (**b**) Impact of residence time (40–120 min) on HTL product distribution.

The effect of operating temperature and residence time on the distribution of product streams during the HTL process of MPR feedstock is illustrated in Fig. 4. As illustrated in Fig. 4(a), the bio crude yield demonstrates a notable increase from 28.83% to 38.85% as the temperature rises from 300 °C to 380 °C, followed by a marginal decline of approximately 4% upon further increasing the temperature to 400 °C. A comparable trend of initial increase, followed by a slight decline beyond a threshold operating temperature, has been observed in other HTL studies too involving carbonaceous wastes^48^, food waste^49^, and mixed waste^50^.

Similarly, when the residence time prolongs from 40 to 80 min, as illustrated in Fig. 4b the bio crude yield increases initially, reaching a peak of at 80 min, followed by a decline as the residence time extends to 120 min. At 80 min, the HTL of MPR feedstock achieved a high bio crude yield of 42.26%, whereas extending the residence time to 120 min led to a decrease of approximately 5% in yield. This observed trend, characterized by an initial rise in bio crude yield up to an optimal residence time and a subsequent shift towards higher gaseous product formation, aligns with previous findings by^42,44,45,51^. Consequently, the operational parameters of 380 °C and a residence time of 80 min were selected for all subsequent analyses, as they yielded the highest bio crude production.

It is clear from the Fig. 4 that the production of gaseous products displays a consistent upward trend across the evaluated ranges of temperature and residence time. The gaseous products yield exhibited an increase from 15.05% to 20.25% with respect to the increase in the operating temperature from 300 to 400 °C. Similarly, a raise in residence time from 40 min to 120 min increased the yield of gaseous product from 18.22% to 22.56%. In contrast, the yield of solid residue decreases markedly, by 23.5% with increasing temperature and 8.99% with extended residence time, reflecting the progression of the reaction in a favourable direction. When the operating conditions shift from 300 to 400 °C and from 40 to 120 min, the proportion of the aqueous phase fluctuates between 28.81% and 38.85% for temperature variation and between 24.00% and 31.05% for changes in residence time. Accordingly, a pattern analogous to that observed with operating temperature is evident with residence time, wherein extending the duration from 30 to 90 min declined the solid residue yields and enhanced the gaseous product yields.

The type of feedstock and operating conditions, such as temperature and residence time, play a vital role in determining the yields of all HTL products^52^. In the present study, the MPR feedstock, composed primarily of plastics and organics fraction along with substantial amounts of paper and textiles fractions, necessitating relatively higher temperatures to facilitate effective fragmentation and degradation reactions. However, any further increase in temperature drives the hydrothermal medium into supercritical settings thereby deviating from the desired subcritical state^53^. The HTL products distribution is greatly impacted by this transition, highlighting how crucial it is to maintain ideal operating temperatures for an effective HTL process. Similarly, consistent with findings from previous studies^54–56^, prolonging the residence time beyond the optimal threshold results in enhanced formation of gaseous products, driven by both secondary and tertiary reactions that occur between hydrothermal medium and components of the feedstock. Consequently, reduced residence times are therefore typically favoured to decrease the energy required for generation of one unit mass of liquid product as well as to optimize bio crude yields.

Further, non-catalytic HTL process of heterogeneous feedstocks often requires harsher temperatures, longer residence times and produces undesirable by-products which reduce the process efficiency. Catalysts address this issue by providing tailored pathways for specific product yields. A variety of catalysts, including metal oxide^57^, zeolite-based^58^ and silica-based, and nanoporous catalysts^59^, have been evaluated for their effectiveness in augmenting bio crude yield. Among these, clay- and silica-based catalysts, being eco-friendly and inexpensive, improve bio crude quality by enhancing distillate distribution, energy recovery, and heating value, while also aids the formation of high-grade solid residues with superior adsorption properties^60–62^.

**Fig. 5:**
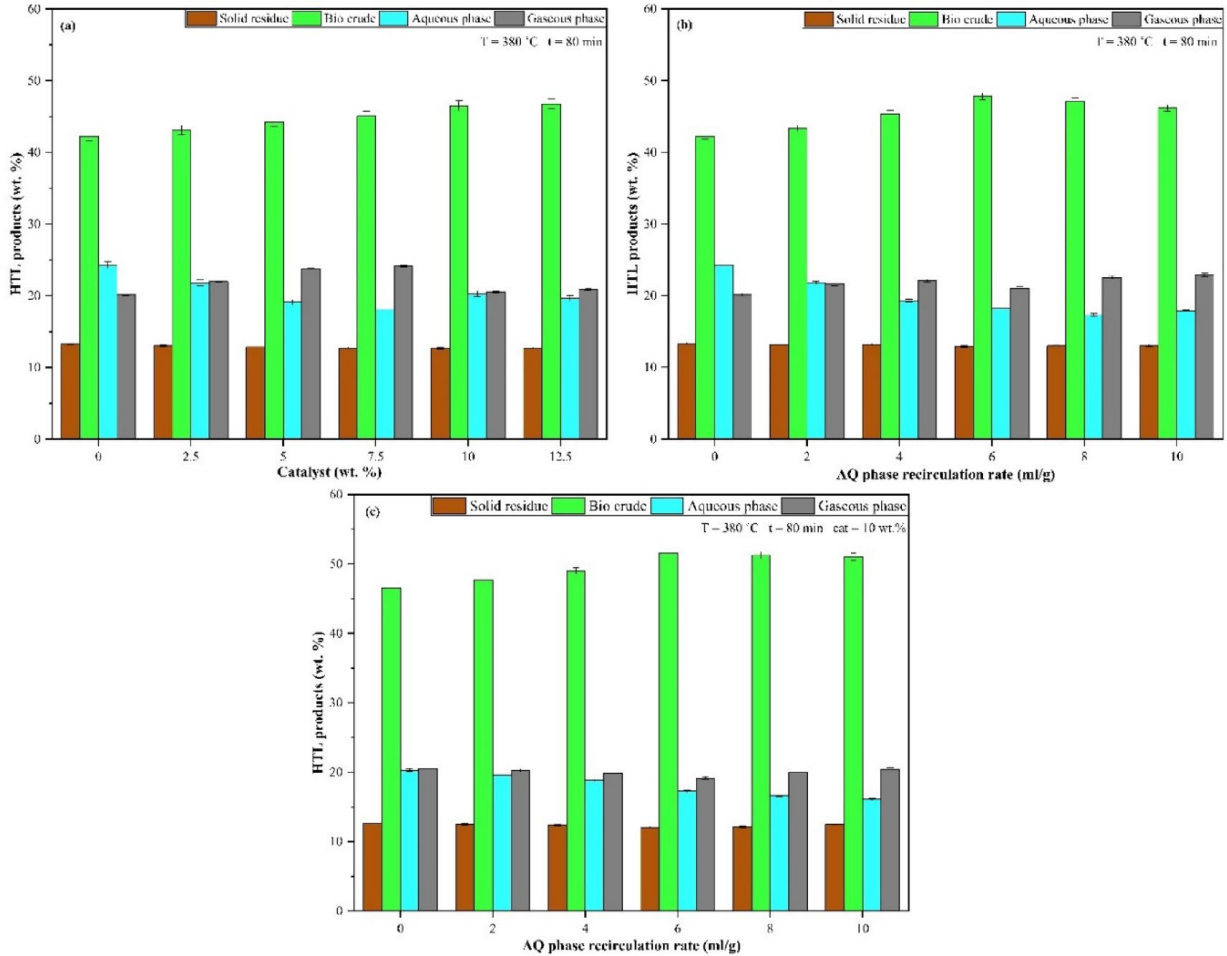
(**a**) Effect of amount of DE catalyst (2.5–12.5 wt%) on yield of HTL products. (**b**) Effect of aqueous phase recirculation (2–10 ml/g) on distribution of HTL products. (**c**) Combined effect of aqueous phase recirculation (2–10 ml/g) and DE catalyst on the yield of HTL products.

Given the heterogeneous feedstock composition, a careful selection of catalysts targeting both food and plastic waste fractions was essential to optimize the process and improve product yield and quality. In the present study, diatomaceous earth (DE) has been utilized as a catalyst. While relatively underexplored, a silica-based catalyst, previous studies have demonstrated its effectiveness in enhancing the yield of liquid products in HTL processes, highlighting its potential and justifying further investigation^21,63^. Figure 5a presents the impact of DE usage on distribution of HTL products.

From Fig. 5a, it is observed that as the catalyst amount increases, the yield in bio crude also rises. The maximum bio crude yield of 46.78% was achieved at 12.5 wt% of diatomaceous earth (DE) catalyst in HTL, which is approximately 10% higher than under non-catalytic HTL conditions. However, when the catalyst amount is intensified from 10 to 12.5% (wt%), the increase in bio crude yield is observed to be minimal, at just 0.6%. Considering both environmental and economic factors, 10 wt% of catalyst was selected as the optimized condition for further HTL investigations of MPR feedstock.

It is worth noting that as the catalyst amount increases, there is a substantial decline in solid residue yield, indicating that the use of the DE catalyst enhances the HTL process by promoting feedstock fragmentation and facilitating decomposition reactions. The yields of products of gaseous and aqueous phases ranged from 20.12% to 24.15% and 18.09% to 24.30%, respectively. Therefore, the addition of a catalyst lowers the yields of solid residue and gaseous products while simultaneously increasing the bio crude yield. Additionally, when organics is hydrothermally liquefied in conjunction with plastics, organics decomposes primarily, and the products that are produced have a favourable impact on the decomposition of plastics by impacting their thermal stability. Furthermore, the HTL products from paper and textile-based organic elements lower the temperature required for plastics decomposition, promoting their depolymerization and the free radicals thus generated interacts with the fragmenting plastic portions, contribute to an overall synergistic effect thereby enhancing the yield of bio crude^17,64^.

Also, in the present work, the aqueous phase generated during the initial HTL runs has been utilized as the hydrothermal medium, replacing pure chemical solvents. This approach aligns with previous studies such as^65–67^, which demonstrated the effectiveness of recirculating the HTL-derived aqueous phase in enhancing product yields. The effect of aqueous phase usage in varying recirculation ratio (RR) such as 2–10 ml/g of feed was investigated in HTL of MPR feedstock and the findings are presented in Fig. 5b.

From Fig. 5b, it is noted that the bio crude yield exhibits an increasing trend with the rise in the aqueous phase recirculation ratio (RR), reaching a peak of 47.78% at 6 ml/g. Beyond this threshold, any further increase in the content of aqueous phase in the hydrothermal medium results in a slight decline of approximately 3% in the bio crude yield. Similarly, the solid residue yield consistently decreases while the gaseous product yield increases, indicating progression of the reaction in the forward direction. The yields in the products of aqueous phase products ranged from 17.85% to 24.3%.

Thus, it can be concluded that, the hydrothermal medium in the HTL procedure significantly affects product distribution by promoting feedstock decomposition reactions. Studies suggest that co-solvents act as stabilizers, mitigating thermal effects by donating hydrogen to neutralize free radicals^68^. Recirculating the aqueous phase offers economic advantages, reduces disposal challenges, and supports a biorefinery concept along with zero-waste approach. The HTL aqueous phase, enriched with organic compounds and often containing toxic compounds like heterocyclic chemicals and phenols, forms a co-solvent denser than water. This reduces water’s dielectric constant, enhancing the solubility of non-polar compounds while preserving sufficient ionic product to favour ionic reactions than radical ones, thereby increasing liquid product yields^53^.

Furthermore, in supposition, the combined impact of catalyst usage and aqueous phase recirculation at different concentrations on HTL of MPR is shown in Fig. 5c. Under aqueous phase recirculated DE catalyst-HTL conditions, the maximum bio crude yield reached 51.60%, representing a 22.10% increase compared to standard HTL, 10.99% higher than DE catalyst-HTL alone (without aqueous phase recirculation), and 7.99% higher than aqueous phase recirculation alone (without DE catalyst). This demonstrates that integrating aqueous phase recirculation with DE catalyst significantly enhances bio crude production. In catalyst-HTL combined with aqueous phase recirculation, the yield of bio crude increases with the concentration of the aqueous phase in the hydrothermal medium, reaching an optimum at 6 ml/g and beyond this concentration, the yield plateaus. Additionally, the solid residue and gaseous product yields reach their lowest levels at 12.05% and 19.12%, respectively, indicating highly favourable reaction conditions. The AQ phase yield remained relatively consistent, ranging from 19.12% to 20.52% across all concentrations of aqueous phase recirculation ratios.

Thus, the use of diatomaceous earth (DE), a mesoporous silica-based catalyst, in combination with the aqueous phase derived from previous HTL experiments of the MPR feedstock, significantly enhances feedstock fragmentation and decomposition reactions. This synergy promotes liquid product formation over gaseous products by increasing the reactivity with respect to macromolecules like polysaccharides, lipids and proteins making it easier for them to participate in reaction pathways^69^. While aqueous phase recirculation alone improves bio crude yield and quality, combining it with DE optimizes feedstock fragmentation, deoxygenation, and dehydrogenation, achieving the highest bio crude yield and quality. This combination also yields the lowest levels of solid residue and gaseous products, underscoring its efficiency in reducing undesired by-products. Notably, the solid residue yield attained under these conditions is the lowest across all experiments, confirming optimal conditions for maximum reaction completion and liquid bio crude production.

### Qualitative assessment of HTL products

The primary and secondary HTL products of MPR, obtained under the conditions of 380 °C for 80 min reaction time through the combined catalytic HTL process incorporating aqueous phase recirculation with the DE catalyst at 10 wt%, and an AQ phase recirculation ratio of 6 ml/g were analysed for their elemental composition and physicochemical properties using ultimate analysis. The elemental compositions for biocrude and solid residues were reported on a dry basis. Since ash and moisture were not separately quantified, oxygen was calculated by difference and may include contributions from inorganic content. Therefore, the oxygen values and derived HHV should be interpreted as indicative rather than ash-free corrected values. This approach is consistent with several HTL studies where ash-free data for products is not routinely reported^50,63,70,71^. Furthermore, process indicative parameters such as the carbon and energy recovery (%) of the HTL products were estimated, with the results detailed in Table 2.

**Table 2:** Elemental analysis of solid residue and bio crude obtained from HTL of MPR.

Sample	HTL Products	C	H	*N*	S	O	H/C	O/C	HHV	CR (%)	ER (%)
MPR Feed		65.72	10.56	0.93	0.26	22.52	1.93	0.26	33.43		
HTL	Bio crude	67.12	11.00	0.9	0.2	20.78	1.97	0.23	34.79	43.16	43.99
	Solid residue	66.06	10.87	0.92	0.21	21.94	1.97	0.25	34.07	13.39	13.58
DE cat - HTL	Bio crude	69.25	11.00	0.89	0.20	18.66	1.91	0.20	35.83	50.34	51.22
	Solid residue	67.25	10.91	0.80	0.20	20.84	1.95	0.23	34.70	13.19	13.38
HTL + AQ	Bio crude	70.66	11.5	0.88	0.18	16.78	1.95	0.18	37.31	49.99	51.89
	Solid residue	69.12	11.25	0.89	0.18	18.56	1.95	0.20	36.16	13.34	13.72
DE + AQ	Bio crude	73.36	12.58	0.80	0.18	13.08	2.06	0.13	40.32	57.59	62.23
	Solid residue	71.89	11.98	0.80	0.18	15.15	2.00	0.16	38.65	13.18	13.93

The carbon percentage (C) of the solid residue and bio crude generated from the HTL of MPR feedstock, under the influence of the catalyst, aqueous phase recirculation, and their combined effects, exhibits an increase of 2.13% to 11.62% compared to the original MPR feedstock. The bio crude that was produced from the DE cat - HTL process with aqueous phase recirculation (DE + AQ) had the greatest carbon constitution (73.36%), with the C content of all the produced bio crudes and solid residues ranging roughly between 66% and 73%. The bio crude from the DE cat and AQ process had the greatest hydrogen percentage (H), at 12.58%, while the other bio crudes had a range of 10.8% to 12.5%. The substantial increase in C and H content can be assigned to the improved fragmentation, facilitating more effective penetration of the hydrothermal medium into the feedstock network. The nitrogen percentage (N) of the bio crude and solid residue obtained ranged between 0.8% and 0.9%. Such low nitrogen levels enhance their suitability for transportation applications and reduce the likelihood of catalyst fouling, thereby simplifying upgrading processes^65,72,73^. The sulphur percentage (S) of all the bio crudes produced remained relatively consistent at 0.18% across the samples.

Among the bio crudes, the physicochemical properties including the H/C ratio and HHV showed the trends: DE + AQ > HTL + AQ > DE cat - HTL > HTL. Conversely, the O/C ratio exhibited an opposite trend. There trends can be ascribed to the circumstance that, the O content of the bio crudes produced approximately ranged from 13% to 22%, with the lowest value of 13.06% observed in the bio crude derived from the DE cat - HTL process of MPR with aqueous phase recirculation. Comparable findings have been reported in studies^74–76^, where bio crude produced through the solvothermal liquefaction process demonstrated improved properties, including higher HHV, H/C ratios, and optimized O/C ratios. Further, the solid residues formed from the HTL process can be considered a valuable carbon source due to their notably high C and H content. Likewise, the bio crudes demonstrated superior qualities, including high H/C, and HHV values, coupled with a low O/C ratio, which simplifies upgrading processes and enhances practical applicability. The percentages of carbon and energy recovery for solid residue followed the trend: DE + AQ > HTL + AQ > DE cat - HTL > HTL.

**Fig. 6:**
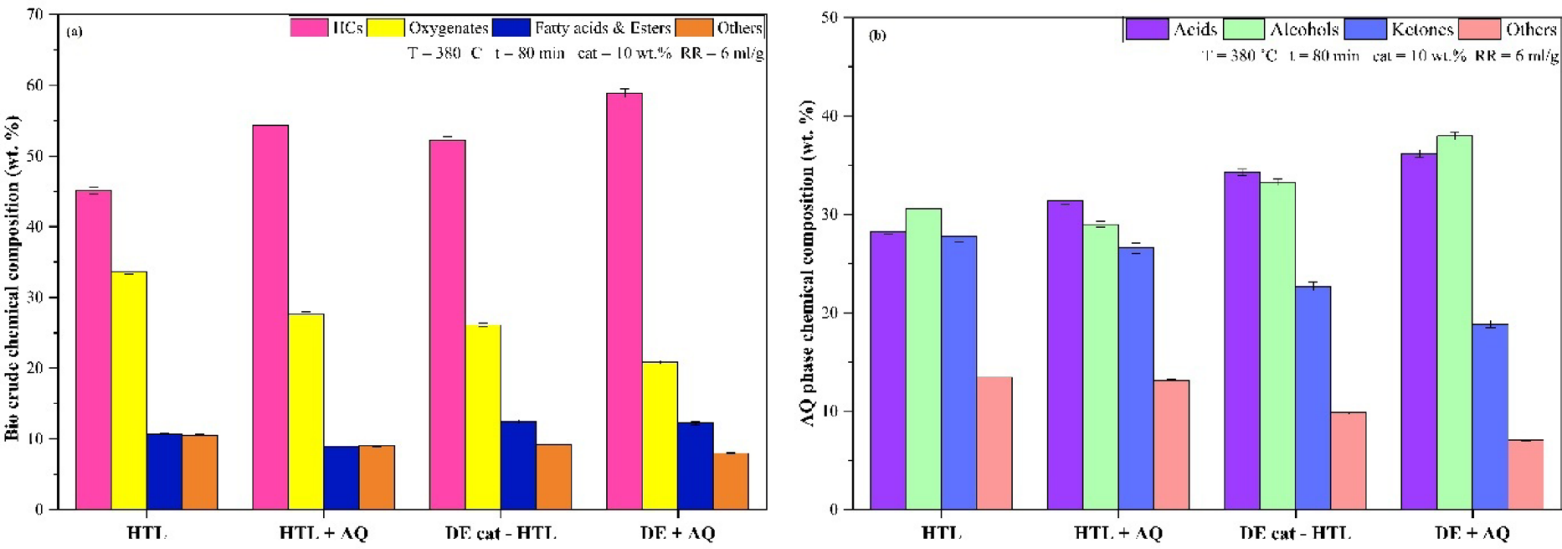
(**a**) GCMS analysis of bio crude. (**b**) GCMS analysis of aqueous phase obtained from HTL of MPR.

Further, GCMS analysis was carried out on the bio crudes generated through HTL, DE cat - HTL, HTL + AQ and combined DE + AQ processes and the findings are presented in Fig. 6(a). The identified organic constituents were categorized into four primary groups: hydrocarbons, oxygenates, fatty acids and esters and others.

The DE + AQ process produced the highest hydrocarbon (HC) fraction, accounting for 58.92%, which represents a 12.8% to 30.6% increase compared to that of DE cat – HTL, HTL + AQ and conventional HTL processes. Similarly, the yields of the oxygenates group as well as esters and fatty acids group exhibited the trend: DE + AQ < HTL + AQ < HTL + AQ < HTL. The ‘Other’ compounds, including nitrogen-containing species, furfurals, and phenols, constituted 8% of the bio crude from DE + AQ, which is 13–24% lower than those produced by other HTL processes of MPR feedstock. The results demonstrate that utilizing diatomaceous earth as a catalyst, in conjunction with AQ phase recirculation, markedly improves the production of target hydrocarbons while minimizing the formation of undesired by-products, including oxygenates and fatty acids. Furthermore, the obtained GCMS results are in concurrence with results from the preceding section, where the bio crude derived from DE + AQ process exhibited the highest quality among all tested HTL conditions.

The decomposition of MPR through various chemical pathways results in a diverse array of bio crude products. The organic component undergoes condensation and conjugated addition reactions, synthesizing aromatic and polycyclic HCs like propylbenzene, fluorene, methylphenanthrenes and benzopyrene. The lipid content in the MPR feedstock contributes to the generation of fatty acids and esters namely penta, octa, hexa-deconic acids, methyl phthalate and esters including 2-hydroxy-1-(hydroxymethyl) ethyl ester, 2,3-dihydroxy propyl ester, and bis (2-Ethylhexyl) ester^77,78^. Additionally, the plastic fraction undergoes cracking and depolymerization, producing long-chain aliphatic hydrocarbons and cyclic compounds like decahydronaphthalene and ethyl-cyclohexane^71^. The textile and paper component, rich in cellulose, contributes oxygenates, such as aldehydes, ketones and secondary alcohols like isoropanol, cyclopentadione, and cyclohexyl ketone, through hydrogenation reactions^16,17^. The breakdown of cellulose, hemicellulose and lignin facilitates the production of furan derivatives (e.g., 3-methyl-2-benzofuranone, 2-furancarboxamide, 2,5-dimethylfuran, Deoxypentofuranose, dihydro-5-methyl furanone, and monomethyl- benzofuran) and phenolic compounds, showcasing the complex interplay of thermal and catalytic processes in bio crude synthesis^79–81^.

Figure 6b illustrates the composition of the aqueous phase generated from the HTL of MPR at 380˚C and 80 min under varying operational conditions.

The identified aqueous phase products are classified into four classes, viz. (i) acids, (ii) alcohols, (iii) aldehydes and (iv) ketones and others. The acid part of the aqueous phase derived from all HTL conditions varied between 28.26% and 36.15%. This can be attributed to breakdown and deamination of organics in MPR, that result in release of organic acids, which acts as catalyst towards bio crude synthesisation at high temperatures^69,82^,

The alcohol portion of the aqueous phase derived from all HTL conditions exhibits significant variation, 28.98% to 37.96% under conditions of 380 °C and 80 min. Fragmented feedstock components undergo alkylation and esterification facilitated by alcoholic solvents, enhancing bio crude production^83^. The remaining aldehydes and ketones along with the “others” portion (comprising phenols and nitrogen containing compounds) ranges from 18.86% to 27.76% and 7.03% to 13.42%, respectively. Minimum values of 18.86% and 7.03% are observed at 380 °C and 80 min, supporting effective aqueous phase recirculation.

Notably, the concentrations of acids and alcohols in the aqueous phase produced by DE cat - HTL and DE + AQ process are nearly equivalent, creating an optimal balance of these components. However, the ketones and “others” fraction concentrations are found to significantly lower in DE + AQ process, making it a more favourable hydrothermal medium alternative to water. The aqueous phase density is recorded at 1.210 g/ml, 21.0% higher than water, facilitating hydrogen radical generation and enhancing process stability, thereby serving as an efficient co-solvent for HTL of MPR.

Furthermore, to differentiate and characterize the crude fractions, boiling point analysis by means of fractional distillation was conducted and the outcomes are presented in Table 3. The bio crude was fractionated into four temperature regimes such as gasoline below 190 °C, diesel between 190 and 340 °C, vacuum oil between 340 and 540 °C and residue above 540 °C. The analysis revealed that bio crude derived from HTL process of MPR feedstocks in the DE + AQ process conditions, exhibits an elevated content of both gasoline and diesel portions with 20.5% distilling within the gasoline range and 31.6% within the diesel range. The presence of 52.1% of the bio crude distilling within 340 °C temperature regime indicates that bio crude derived from HTL of MPR feedstock under DE + AQ conditions is well-suited for potential use in transportation applications. These findings closely align with those reported by^38,39,63,84^. The 25.5% of crude found in the residue fraction is largely due to the substantial presence of large plastic polymers, which comprised the majority of the MPR feedstock. This observation is further supported by the detection of unreacted plastic residues adhering to the agitator blades at the conclusion of each HTL experimental run.

**Table 3:** Boiling point analysis results of bio crude under DE + AQ conditions.

Type	Range of Temperature	% fraction
Gasoline	less than 190 °C	20.5
Diesel	190–340 °C	31.6
Vacuum oil	340–540 °C	22.4
Residue	greater than 540 °C	25.5

**Fig. 7:**
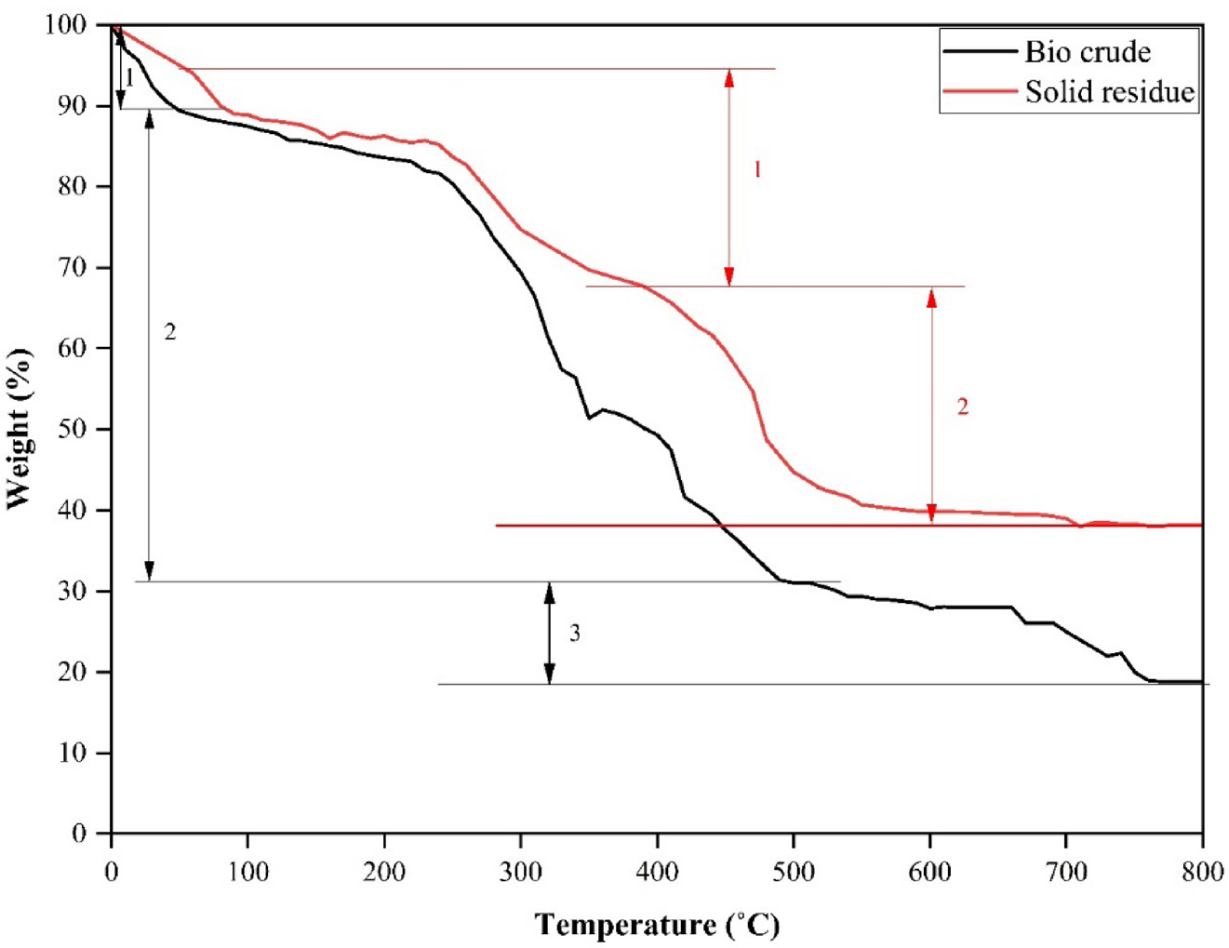
TGA results of bio crude and solid residue.

Also, TGA assessment was performed to analyse the thermal stability of the bio crude and solid residue obtained from HTL of MPR under DE + AQ conditions. The results concerning TGA is shown in Fig. 7. It is evident that, the bio crude distinctly undergoes three separate phases of thermal degradation: an initial low-temperature thermal decomposition phase between 25 °C and 80 °C, peaking at approximately 55 °C, a mid-temperature thermal decomposition phase spanning from 150 °C to 520 °C, with a peak at 350 °C, and a high-temperature decomposition phase from 500 °C to 750 °C, characterized by a peak at 680 °C. The first phase (region 1) is characterized by the moisture evaporation and decomposition of lower molecular weight organic acids and oxygenates. The intermediate phase (region 2) involves the volatilization of lower to medium molecular weight organic compounds, while the final phase (region 3) pertains to the degradation of medium to higher molar mass organic materials.

Similarly, the TGA of solid residue formed from HTL process of MPR under DE + AQ conditions, exhibits two separate thermal degradation phases: a broader low-temperature phase between 100 °C and 350 °C, peaking at 250 °C, and a more pronounced high-temperature range from 400 °C to 720 °C, peaking at 450 °C. The initial phase is associated with the release of medium to a higher molar moss compounds in relatively lower mass proportions. In contrast, the high-temperature phase close to 470 °C is attributed to the presence of residual unreacted plastic waste from MPR feedstock being retained in the solid residue after the HTL process. Also, it is important to note that, the TGA residual mass (15% for biocrude and 35% for solid residue) represents the sum of thermally stable carbonaceous char, inorganic ash, and non-volatile/unconverted fractions.

**Fig. 8:**
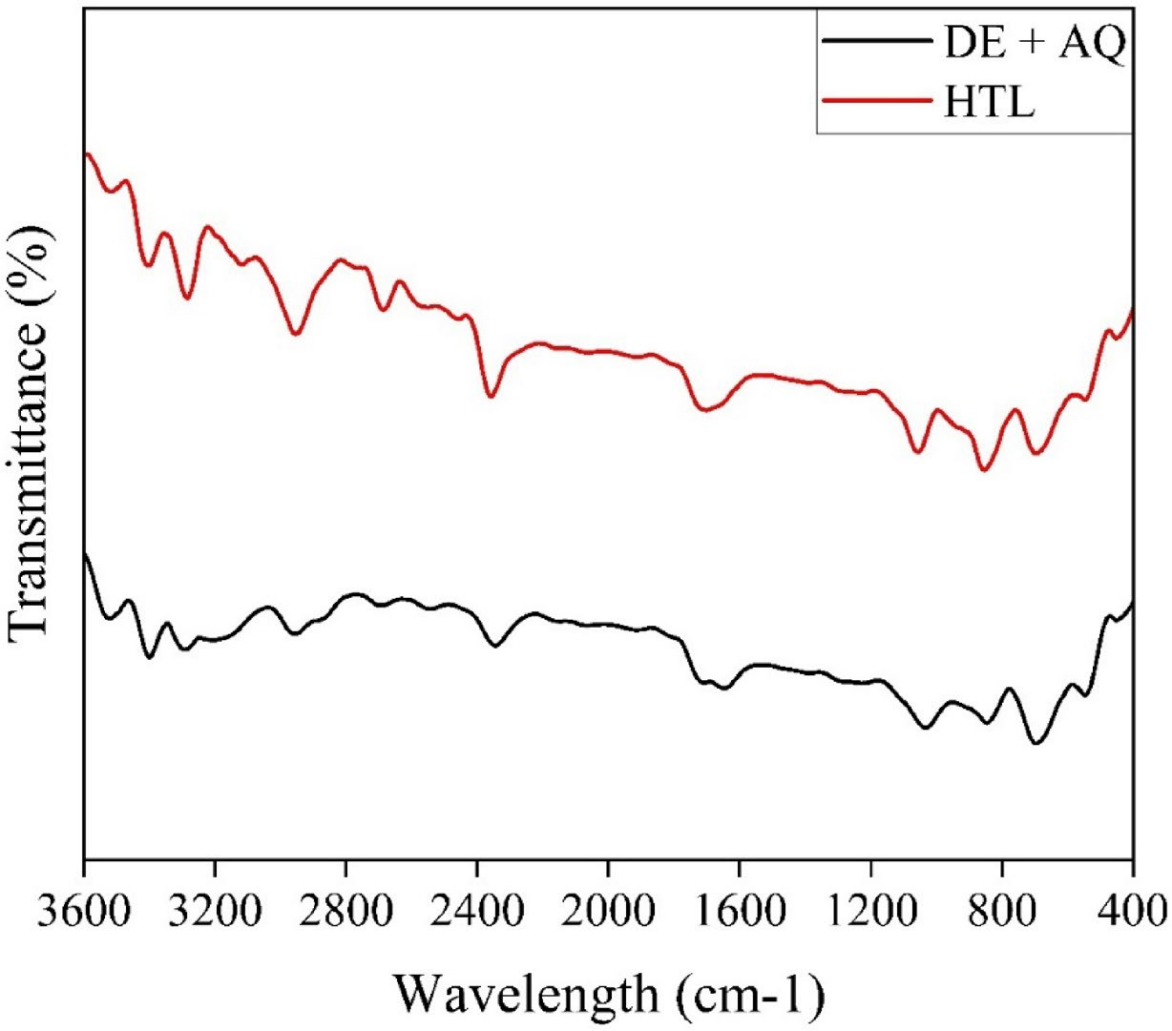
FTIR results of solid residue.

Also, the solid residue produced from DE + AQ and HTL process were analysed using Fourier-transform infrared (FTIR) spectroscopy to recognize the functional groups and metal contents. From both the curves of Fig. 8, it can be seen that, peak between 3400 cm⁻¹ and 3550 cm⁻¹ corresponds to –OH stretching, that can be attributed to absorbed water, alcohols, and carboxylic acids, derived from lipid compounds in organics and the depolymerization of plastics. Prominent N–H and C–H stretching bands, identified at 3000 cm⁻¹ and 2700 cm⁻¹, respectively, are attributed to nitrogen-containing compounds from organic and textile waste, as well as by-products generated during the cracking of plastic waste.

Also, peaks around 2400 cm⁻¹ suggest the presence of alkynes (C ≡ C), nitriles (C = N), and aldehydes (C = C) derived from hemicellulose in organic and paper waste subcomponents. Additional peaks between 1600 cm⁻¹ and 1750 cm⁻¹ indicate C = C and C = O stretching, representing conjugated acids, benzene rings, aldehydes, and ketones derived from the cellulose present in textile and paper waste. Bands around 1110 cm⁻¹ and 1040 cm⁻¹ correspond to C–O and Si–O–Si stretching vibrations due to primary alcohols, resulting from initial decomposition and depolymerization reactions and the presence of residual catalyst. Further, a distinct peak at 545 cm⁻¹ confirms the presence of Si–O–Si bonds, confirming DE residual catalyst retention in the solid residue. This finding highlights the potential of these residues as recyclable catalysts for future processes, contributing to both the economic efficiency and sustainability of the biorefinery system.

**Fig. 9:**
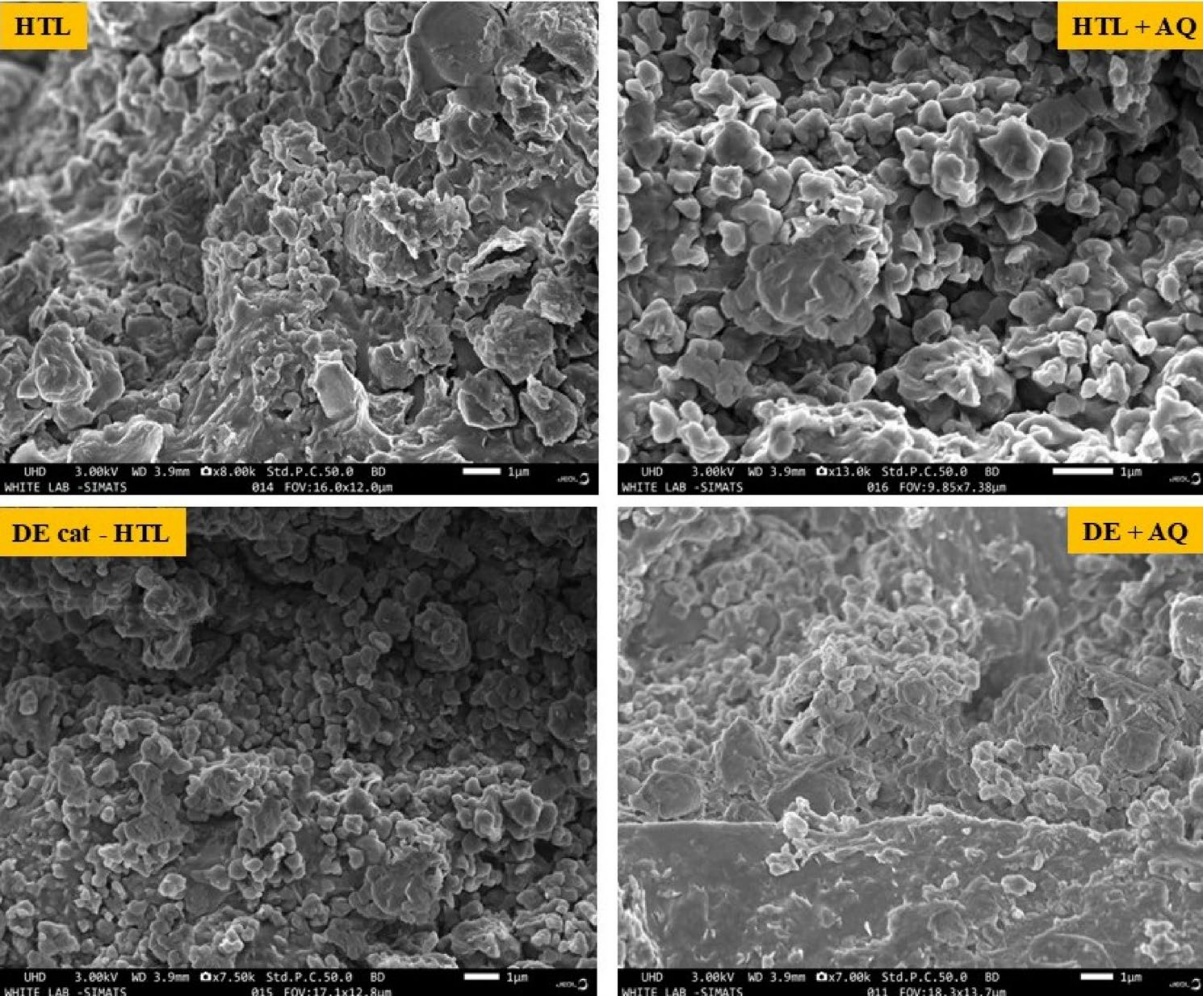
FESEM results of solid residue obtained from HTL of MPR sample.

**Fig. 10:**
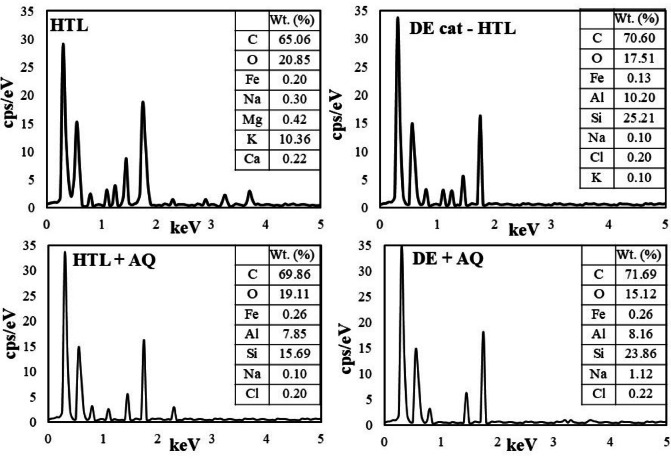
EDS results of solid residue.

Furthermore, Figs. 9 and 10 shows the results of FESEM and EDS analysis (to examine the surface characteristics) of solid residues generated from various hydrothermal liquefaction (HTL) processes of MPR feedstock.

At a 1 μm length scale, the FESEM images reveal that, residues from non - catalytic HTL processes such as normal HTL and HTL + AQ processes display highly irregular pore shapes and a wide range of pore sizes, with average pore diameters measuring 122.52 nm and 100.96 nm, respectively. In contrast, solid residues from cat-HTL and DE + AQ processes displayed comparatively less irregular pore shapes and narrower pore size distributions. At the same resolution, the average pore sizes were larger, measured at 156.89 nm for cat-HTL and 175.36 nm for DE + AQ, indicating the influence of catalytic and modified process conditions on the pore structure.

The degradation of moisture and volatiles in solid residue leads to the formation of pores. The majority of organic compounds deposited on the surface are eliminated during heat treatment through thermal decomposition and their subsequent removal from the solid residue surface^85^. Additionally, the images from FESEM analysis highlight significant formation of agglomerates, likely caused by fine inorganic particles interacting under extreme operating conditions to form aggregation complexes^85^. Solid residues produced through HTL processes exhibit a rougher and more fragmented structure due to the effects of the liquefaction medium^86^. These solid residues are being extensively studied for their potential as adsorbent in wastewater treatment and various engineering applications due to their innate surface characteristics^87–89^.

The EDS analysis of solid residues from non-catalytic HTL process, presented in Fig. 10, revealed an elevated presence of carbon (C) and oxygen (O), along with moderate levels of sodium (Na), chlorine (Cl), Ferrous (Fe), Magnesium (Mg) and potassium (K). These Na, Cl, and K impurities are hypothesized to originate from the MPR feedstock. In contrast, solid residues from DE cat - HTL and DE + AQ processes showed the presence of aluminium (Al) and silicon (Si), corroborating the findings from the FTIR analysis on the presence of residual catalyst in solid residues after cat – HTL processes. Additionally, the obtained EDS results align with the CHNS elemental analysis results of solid residues observed in all HTL experiments reported in the Sect. 3.3.1.

### Mechanistic interpretation

To rationalize the experimental trends in bio crude yield, elemental composition, and product distribution, a mechanistic interpretation of the hydrothermal liquefaction (HTL) process was developed based on the analytical evidence obtained in this study. The trends observed in product yield, elemental composition, and molecular distribution provide insight into the underlying chemical transformations during hydrothermal liquefaction (HTL) of marine pollutant residues (MPR). The combined use of diatomaceous earth (DE) catalyst and aqueous-phase (AQ) recirculation resulted in the highest bio crude yield (≈ 51.6%) and energy content (≈ 40 MJ kg⁻¹), accompanied by a lower O/C ratio and higher H/C ratio relative to non-catalytic runs. FTIR spectra showed diminished carbonyl (C = O) and hydroxyl (O–H) stretching peaks, while GC–MS analysis revealed increased long-chain hydrocarbons and fewer oxygenated compounds. These compositional shifts indicate effective deoxygenation, selective cracking, and improved hydrocarbon formation under DE and AQ-modified conditions.

The silica-rich DE catalyst contains surface silanol (Si–OH) groups that act as mild acidic sites, facilitating dehydration, decarboxylation, and cracking reactions^90,91^. These reactions promote the conversion of oxygenated intermediates and long-chain polymer fragments into lower molecular weight hydrocarbons, releasing H₂O and CO₂ as by-products. The catalytic steps can be represented schematically as Eqs. (6) and (7).

6 $$R - C{H_2} - CH(OH) - {R^\prime }\mathop \to \limits^{{DE~(Si - OH)}} R - CH=CH - {R^\prime }+{H_2}O$$

7 $$R--COOH~~~~~~~\mathop \to \limits^{{DE}} ~~~~~~R--H+C{O_2}$$

Simultaneously, the recirculated aqueous phase—rich in low-molecular-weight organics such as alcohols, ketones, and carboxylic acids—acts as a hydrogen-donating co-solvent and radical stabilizer^92–94^. These species enhance hydrogen transfer reactions, mitigating secondary condensation and polymerization that lead to char. Representative reactions are represented by Eqs. (8) and (9).

8 $$R \bullet ~+~{R^\prime } - OH \to R - H~+~{R^\prime } \bullet ~$$

9 $$R \bullet ~+~{R^\prime } \bullet \to R - {R^\prime }$$

The synergy between DE and AQ recirculation arises from the concurrent promotion of oxygen removal and hydrogen addition. While DE enhances C–O bond cleavage and oxygenate conversion, the AQ phase provides a reductive environment that stabilizes reactive intermediates. This dual effect favors liquid-phase condensation and alkylation pathwaysover solid-phase polymerization, resulting in increased bio crude yield and improved quality.

Overall, the reaction sequence can be summarized as:

thermal depolymerization → catalytic dehydration/decarboxylation → hydrogen transfer and radical stabilization → condensation and hydrocarbon enrichment → phase separation into oil, aqueous, and solid fractions.

### Sustainability and practical implications

A rigorous sustainability evaluation of hydrothermal liquefaction (HTL) of MPR feedstock requires quantification of process-level energy flows, material recovery and operational limitations. In this study, the sustainability assessment was steered by the experimental data on product yields, elemental recovery and heating values, and quantitative metrices such as Net Energy Ratio (NER), carbon recovery, and aqueous-phase recirculation behavior. Under optimized DE + AQ conditions, the system achieved a bio crude yield of 51.6% with an HHV of 40.3 MJ kg^-1^, accompanied by a solid residue HHV of 38.6 MJ kg^-1^.

#### Energy performance and net energy ratio (NER)

Using the detailed thermodynamic calculations presented in the Supplementary Information, the total recoverable energy from the liquid and solid products under optimized DE + AQ conditions was 25.46 MJ kg^-1^ of feedstock. The corresponding process energy input was quantified by accounting for the total electrical energy consumption of the three operating units—heater, pump, and mechanical stirrer—during both the heating (ramp) period and the isothermal reaction stage. For the heating phase, the ramp time was calculated based on the enthalpy required to raise the MPR feedstock, the fresh water, and the recirculated aqueous phase from ambient temperature to 340 °C under a pressure of 25 MPa. The enthalpy of the AQ phase was treated within a theoretical framework to evaluate how recirculating the aqueous phase at progressively higher temperatures would influence the overall heat demand of the system and thereby affect the process level energy balance. Electrical energy consumption was then computed using the rated powers of the equipment throughout their respective operating durations. This integrated approach allowed the estimation of total process energy input for multiple AQ temperature scenarios, enabling a more accurate evaluation of NER under different heat integration conditions. The resulting NER values, tabulated in Table 4, ranged from 1.02 to 5.74 as the AQ recirculation temperature increased from 25 to 300 °C, indicating that the system transitions from marginally energy-positive to strongly energy-positive when enthalpy rich AQ streams are utilized. These results show that the HTL system becomes increasingly energy-favorable when thermal integration or industrial waste-heat recovery is applied. However, under standard laboratory conditions with cold AQ recirculation, the NER remains close to unity, and therefore cannot be used to claim, with certainity, inherent or unconditional sustainability of the process.

**Table 4:** Calculated NER values at different temperatures.

Temperature (ºC)	Energy output from the system (E_out_)	Energy Input from the system (E_in_)	NER
25	25.46	25.34	1.00
50	25.46	23.67	1.08
100	25.46	20.18	1.26
150	25.46	16.97	1.50
200	25.46	13.48	1.89
250	25.46	9.87	2.58
300	25.46	5.95	4.28

#### Resource efficiency and carbon recovery

Carbon distribution analysis showed that ~ 70% of the feed carbon is retained in the bio crude and ~ 10% in the solid residue under DE + AQ conditions, values consistent with prior subcritical HTL studies on mixed wastes^63,71^. These results confirm that the process effectively channels carbon into usable energy carriers. However, unconverted plastic fragments detected in residues indicate incomplete depolymerization, signalling the need for further optimization of reaction conditions for full material circularity.

#### Circularity and process constraints

Indicated by the results from the study, the recirculated aqueous phase (AQ) contains organic acids, alcohols, ketones, and low-molecular-weight intermediates that enhance hydrogen donor availability and contribute to improved reaction selectivity, of solvent HTL systems. Also, DE catalyst retention within the solid residue suggests potential for catalyst recycling. However, several operational constraints limit the degree of achievable circularity. Marine-derived feedstocks introduce salts, metals, and halogens known to accelerate reactor corrosion and alter catalytic behaviour^95,96^. Additionally, the AQ phase contains phenolic and nitrogenous compounds with documented aquatic toxicity, requiring post-treatment prior to disposal or reuse^72,97^. These constraints indicate that although AQ recirculation enhances energy performance, it does not eliminate the environmental burdens associated with the process.

#### Practical implications for deployment

The results demonstrate that HTL can effectively valorize heterogeneous marine pollutant residues, provided optimized operating conditions and thermal integration strategies are employed. Potential deployment scenarios include coastal waste-collection centers, port-based waste-handling units, and systems where high-moisture mixed wastes and available waste heat streams coexist. However, large-scale implementation must consider heat losses, pump and compressor inefficiencies, corrosion management, and aqueous-phase treatment costs. Therefore, while the process shows potential applicability, it should not be interpreted as fully sustainable without comprehensive life-cycle, economic, and emissions assessments.

Overall, the process represents a practical circular solution for coastal waste management by transforming marine plastic residues into renewable energy carriers. The integration of catalyst reuse, aqueous-phase recycling, and moderate operating conditions reinforces the process’s applied scalability and alignment with Sustainable Development Goals 7 (Affordable and Clean Energy) and 14 (Life Below Water).

## Conclusion

The present study evaluated hydrothermal liquefaction (HTL) of marine pollutant residues (MPR) using diatomaceous earth catalysis and aqueous-phase recirculation. Under optimized conditions (380 °C, 80 min, 10 wt% DE, RR = 6 mL g⁻¹), the process produced a maximum bio crude yield of 51.6% with high heating values and favorable H/C ratios. GC–MS analysis confirmed enhanced hydrocarbon generation, while TGA, FTIR, FESEM, and EDS characterized the stability and composition of associated solid residues. Energy analysis showed that the system can achieve NER values above unity, particularly when recirculated aqueous-phase enthalpy contributes to reduced external heating demand. These energy and material indicators demonstrate the process’s potential for valorizing heterogeneous marine residues. However, the sustainability of HTL at scale remains conditional on thermal integration, management of salt and contaminant loadings, aqueous-phase treatment, and reactor durability. The findings therefore provide a technical foundation for further investigation rather than a complete sustainability validation of the process, supporting circular economy practices and Sustainable Development Goals 7 and 14, by converting marine pollution residues into value-added energy resources.

## Supplementary Information

Below is the link to the electronic supplementary material.

Supplementary Material 1

## Data Availability

The datasets used and/or analysed during the current study available from the corresponding author on reasonable request.
