# Supplementary material for: Sustainable valorization of marine plastic residues via hydrothermal liquefaction for clean energy recovery
Source: Sci Rep. 2026 Jan 13;16:2755. doi: 10.1038/s41598-025-32471-3 (PMC12824407; doi:10.1038/s41598-025-32471-3)
Supplement: Supplementary file 1 — Supplementary Material 1 [file 41598_2025_32471_MOESM1_ESM.docx]

**Sustainable Valorization of Marine Plastic Residues via Hydrothermal Liquefaction for Clean Energy Recovery**

**Mahadevan Vaishnavi, S.Raja*1, Maher Ali Rusho2 , Tesfaye Barza Zema**3**

1* Center for Advanced Multidisciplinary Research and Innovation, Chennai Institute of

Technology, Chennai, Tamilnadu, India-600069, (sraja@citchennai.net) 1

2 Masters of Engineering in Engineering Management, Lockheed Matin Engineering

Management, University of Colorado, Boulder, Colorado-80308; maru4732@colorado.edu

3**College of Engineering, Division of Mechanical Engimeering, Wolaita Sodo

University, 109-2244 Sst Sodo, Ethiopia

(Corresponding author(s): sraja@citchennai.net ; tesfayebarza@wsu.edu.et)

**Supplementary Information**

**Assumptions – Net Energy Ratio Calculation**

- NER is defined as

$$\mathrm{NE}R_{\mathrm{proc}}=\frac{E_{\mathrm{out}}}{E_{\mathrm{in},\mathrm{process}}}$$

where $E_{\mathrm{out}}$= chemical energy in useful products (HHV basis), and $E_{\mathrm{in},\mathrm{process}}$= externally supplied process energy (electrical/thermal) required to operate the system. The feed HHV (biomass chemical energy) is not included in $E_{\mathrm{in},\mathrm{process}}$because the feed is waste biomass with negligible upstream energetic burden. Energy outputs included liquid biocrude and solid residue energy (HHV × mass).

- The following values were employed.

MPR feedstock basis = 1 kg

Biomass : hydrothermal medium = 1:10

Quantity of hydrothermal medium = 1000 ml

AQ phase recirculation ratio = 6 ml/g

Quantity of recirculated aqueous phase = 600 ml

Quantity of fresh water = 400 ml

Biocrude yield = 51.60 wt%

Solid residue yield = 12.06 wt%

HHV of biocrude = 40.32 MJ/kg

HHV of solid residue = 38.65 MJ/kg

Initial temperature of the reactor contents = 25 ºC

Operating Temperature = 340 ºC

Operating Pressure = 25 MPa

Reaction / Holding time = 80 min

Heater rated power = 5 KW

Heater useful fraction = 0.75

Pump power rating = 0.5 HP = 0.373 KW

Stirrer power rating = 0.5 HP = 0.373 KW

- The thermodynamic properties of water were obtained from standard NIST handbook.
- The specific heat of MPR feedstock was obtained from literature (1.5 KJ/kg.K).
- The enthalpy of the aqueous phase is assumed to be equal to that of fresh water at 25 MPa.
- No phase change was assumed for water because $P>P_{\text{crit}}$(25 MPa > 22.064 MPa) and $T_{f}<T_{\text{crit}}$, thus water is treated as single phase.
- No or negligible heat loss was assumed during reaction / hold time.

**Energy output from HTL system**

$Amount of bio crude =\frac{51.6}{100}*1000=516 g=0.516 kg$

$$Energy output from bio crude=0.516 kg*40.32\frac{MJ}{kg}=20.81MJ$$

$$Amount of bio crude =\frac{12.05}{100}*1000=120.5 g=0.1205 kg$$

$$Energy output from solid residue=0.1205 kg*38.65\frac{MJ}{kg}=4.66 MJ$$

$$Total energy output=energy output from bio crude+energy output from soild residue$$

$$\boldsymbol{Total energy output from HTL process=25.46 MJ}$$

**Energy input to HTL system**

- Amount of heat required for heating MPR feedstock

$$Q_{MPR feedstock}=mC_{p}\Delta T$$

$$Q_{MPR feedstock}=1 kg*1.5 \frac{KJ}{kg.˚K}*\left( 340-25 \right)˚K=472.5 KJ=0.4725 MJ$$

- Amount of heat required for heating hydrothermal medium

$$Q_{HTL medium}=m\Delta h$$

$$Q_{HTL medium}=10000 ml*1 \frac{g}{ml}*\frac{1}{1000}*\left( 1594.5-105.1 \right)\frac{KJ}{kg}=14894 KJ=14.894 MJ$$

- Total heat required for heating feed for HTL process

$$Q_{feed}=Q_{MPR feedstock}+Q_{HTL medium}=15.367 MJ=4.27 KWh$$

- Heat energy available

Heater rated capacity = 5 KW

Heat energy available = 5 * 0.75 = 3.75 KW

- $Heating time required=\frac{4.27}{3.75}=1.14 h=68.3 min=4099.2 sec$
- Energy consumed by electrical equipment during heating time can be calculated as follows
- $Energy consumed by heater=5 KW=5\frac{Kj}{s}=5*4099.2=20496 KJ=20.496 MJ$
- $Energy consumed by stirrer=0.373 KW=0.373*4099.2=1529 KJ=1.529 MJ$
- $Energy consumed by pump=0.373 KW=0.373*4099.2=1529 KJ=1.529 MJ$
- $Total energy consumed by the electrical equipments during ramp time=20.496+1.529+1.529=23.554 MJ$
- Further, during the 80-minute holding (reaction) period, ideal adiabatic conditions are assumed—i.e., the reactor is considered perfectly insulated with no heat loss to the surroundings. Under this assumption, the target reaction temperature of 340 °C is maintained throughout the holding time without any additional external heat input. Any potential heat losses are offset by the heat generated from the ongoing reactions within the reactor. Consequently, only the stirrer operates during this period. Therefore, the electrical energy consumption associated with the heating phase can be calculated as follows:

$$Energy consumed by stirrer=0.373 KW=0.373*80*60=1790.4 KJ=1.79 MJ$$

$$\boldsymbol{Total energy}\boldsymbol{in}\boldsymbol{put}\boldsymbol{to}\boldsymbol{HTL process=25.}\boldsymbol{34}\boldsymbol{MJ}$$

- **Thus, NER is calculated to be 1.00**

Using the same methodology, the NER values for the different cases of aqueous-phase recirculation temperature were calculated and are presented in the main manuscript. It should be noted that, as the temperature of the recirculated aqueous phase increases, its enthalpy correspondingly increases. As a result, the external heat required to achieve the desired reaction temperature decreases, leading to an improvement in the overall NER of the process.
